# Duodenal Wedge Resection for Large Gastrointestinal Stromal Tumour Presenting with Life-Threatening Haemorrhage

**DOI:** 10.1155/2013/562642

**Published:** 2013-03-27

**Authors:** Alexander Shaw, John Jeffery, Laura Dias, Sarfraz Nazir

**Affiliations:** ^1^Department of Plastic Surgery, Wexham Park Hospitals, Slough SL2 4HL, UK; ^2^Department of General Surgery, Oxford University Hospitals NHS Trust, Horton General Hospital, Banbury OX16 9AL, UK; ^3^Department of Urology, Wexham Park Hospitals, Slough SL2 4HL, UK; ^4^Department of Radiology, Oxford University Hospitals NHS Trust, Horton General Hospital, Banbury OX16 9AL, UK

## Abstract

*Background*. Duodenal gastrointestinal stromal tumours (GISTs) are an uncommon malignancy of the gastrointestinal (GI) tract. We present a case of life-threatening haemorrhage caused by a large ulcerating duodenal GIST arising from the third part of the duodenum managed by a limited duodenal wedge resection. *Case Presentation*. A 61-year-old patient presented with acute life-threatening gastrointestinal bleeding. After oesophagogastroduodenoscopy failed to demonstrate the source of bleeding, a 5 cm ulcerating exophytic mass originating from the third part of the duodenum was identified at laparotomy. A successful limited wedge resection of the tumour mass was performed. Histopathology subsequently confirmed a duodenal GIST. The patient remained well at 12-month followup with no evidence of local recurrence or metastatic spread. *Conclusion*. Duodenal GISTs can present with life-threatening upper GI haemorrhage. In the context of acute haemorrhage, even relatively large duodenal GISTs can be treated by limited wedge resection. This is a preferable alternative to duodenopancreatectomy with lower morbidity and mortality but comparable oncological outcome.

## 1. Background

Gastrointestinal stromal tumours (GISTs) are generally considered to be an uncommon soft tissue malignancy of the gastrointestinal tract. They predominantly occur in patients over the age of 50. They constitute 0.1% to 3% of all gastrointestinal tumours with an incidence equivalent to 10–20 cases per million [1, 2]. These tumours occur anywhere along the length of the gastrointestinal tract with the majority, 60%–70%, arising in the stomach. A further 20%–30% occur in the small bowel. On the whole GISTs of the duodenum are only found in 3%–5% of cases and it remains a rare location for their development [3]. When they do occur, the majority localise to the second portion of the duodenum (42/156 in one study) and as a result are frequently located close to the ampulla of Vater [3, 4]. Of all those that are surgically resected, 6%–21% are located in the duodenum [5, 6]. 

GISTs tend to present with vague nonspecific abdominal symptoms such as pain, a mass, or an occult gastrointestinal bleed. Occasionally patients will present with more severe symptoms such as obstruction, perforation, or severe acute bleeding as in the case described below [7]. The identification of the source of bleeding may be determined by upper gastrointestinal endoscopy when the tumour is located in the stomach or proximal duodenum. Those found incidentally tend to be small, with a mean diameter of 1.5 cm and carry a better prognosis [8]. Complete surgical resection remains the mainstay of treatment despite recent medical advances and carries an overall 5-year survival rate of 45% (21% to 88%) depending on the tumour grade and completeness of resection [3, 9]. 

## 2. Case Presentation

A 61-year-old man presented to the emergency department with hypovolaemic shock following a sudden episode of upper gastrointestinal bleeding associated with melaena, haematemesis, and a temporary loss of consciousness. There was no prior history of gastrointestinal disturbance, and his past medical history included essential hypertension controlled by antihypertensives. 

On admission to accident and emergency, he was haemodynamically unstable with refractory hypotension (89/60 mmHg) and a sinus tachycardia of 110 bpm. Physical examination revealed melaena on rectal examination and a Glasgow coma score of 14/15 but was otherwise unremarkable. He remained haemodynamically unstable despite aggressive fluid resuscitation with Hartmann's and packed red blood cells. His Rockall score was 3 out of 7, scoring one for age and two for shock.

Initial blood tests revealed a haemoglobin of 9.0 g/dL, and emergency oesophagogastroduodenoscopy (OGD) demonstrated fresh ongoing bleeding with no identifiable source. His haemoglobin continued to drop despite being transfused a total of five units of blood, and inotropic support was needed to maintain his blood pressure and so arrangements were made for emergency surgery.

**Figure 1:**
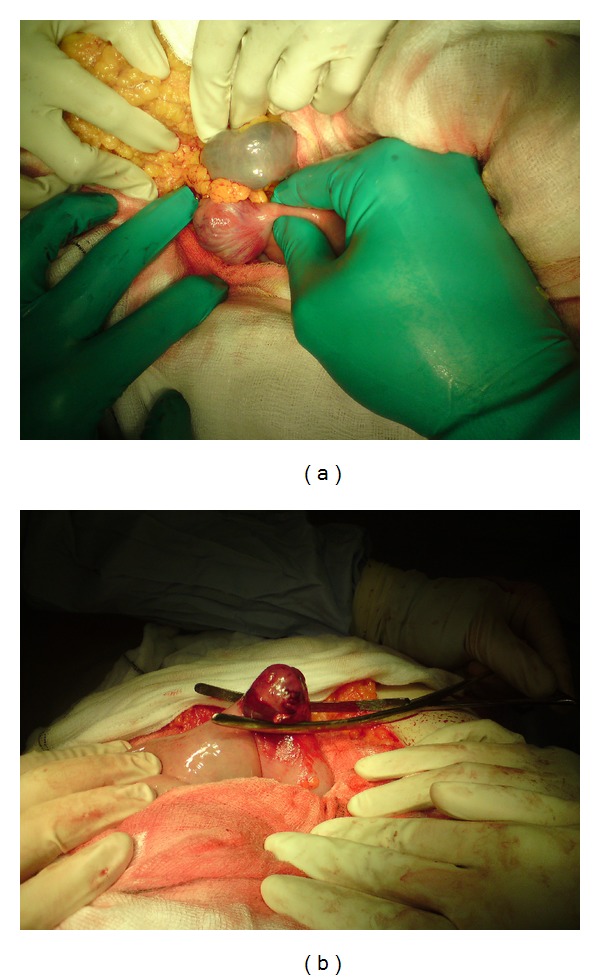
(a) shows intraoperative photograph of haemorrhagic exophytic anterior wall duodenal GIST. (b) demonstrates a clamp across the base of the tumour for intraoperative evaluation of bleeding cessation with the use of endoscopy. Local limited wedge resection was subsequently performed with clear margins. Surrounding bowel can be seen to be healthy allowing for a primary anastomosis.

Emergency laparotomy identified a mass lesion on the anterior duodenal wall between the 3rd and 4th part of the duodenum (D3 and D4, resp.) (Figure 1). Continued haemorrhage was evident from the nasogastric tube, and a bowel clamp applied under the mass with an on-table OGD confirming haemostasis (Figure 1(b)). A duodenal wedge resection was performed to remove the vascular mass followed by primary transverse closure. Two drains were inserted: one abdominal drain and the second intraluminal proximal to the level of the anastomosis to drain descending gastric and pancreatic fluid. A wedge resection was chosen in preference to a more radical surgical procedure as there was no evidence of extension of the lesion beyond the serosa or the involvement of adjacent tissues. The relatively low risk of intramural spread that is characteristic of GISTs was also considered.

In total, he was transfused 12 units of packed red blood cells and 6 units of fresh frozen plasma with one unit of cryoprecipitate and pooled platelets and after surgery he was transferred to intensive therapy unit (ITU). He was extubated after one day and remained haemodynamically stable, not requiring any further transfusions. He was discharged to the ward after 3 days. 

**Figure 2:**
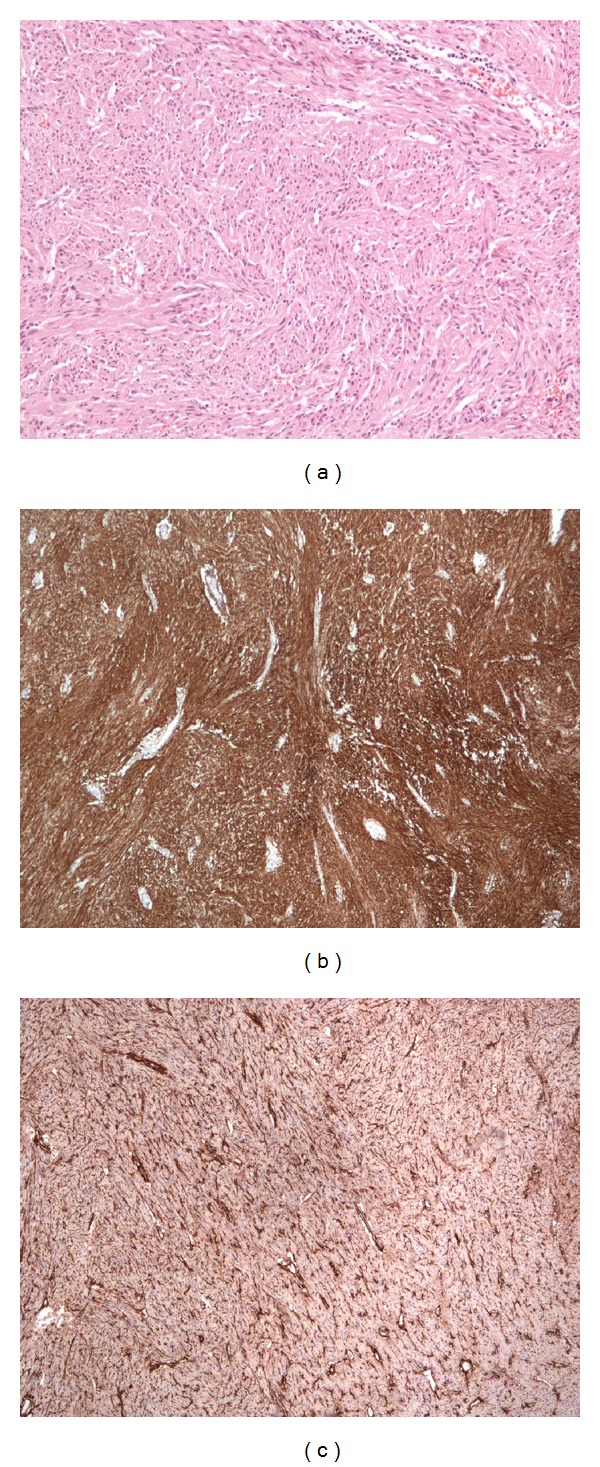
Immunohistochemistry at microscopic level of GIST neoplasm. (a) demonstrates the 10x image with hematoxylin and eosin staining. The tumour had a mitotic index of 2/50 high power fields (HPF). (b) shows positive staining for CD117 (c-kit) which is characteristic of GIST tumours and (c) shows the tumour also staining positive for CD34.

Histopathological analysis of the resected specimen showed a well-demarcated small-bowel tumour measuring 50 × 29 × 28 mm. Clear margins were demonstrated over 2 mm from the stapled resection edge with no mucosal infiltration. Microscopically (Figure 2), it showed strong positive staining for both CD117 (c-kit) and CD34, all being consistent with a gastrointestinal stromal tumour (GIST). 

Staging computerised tomography (CT) revealed no evidence of metastases and he was discharged from hospital after twelve days with 6 monthly followup.

## 3. Discussion

In 1983, Mazur and Clark first described gastrointestinal stromal tumours (GIST) as neoplasms neither showing immunohistochemical or ultrastructural characteristics of neuronal Schwann cells or smooth muscle cells [3]. Prior to this, many believed that GISTs originated from smooth muscle because of their variable staining of muscle markers or even neuronal or myenteric origin because of antibody staining for S-100 protein [7]. Histological examination nowadays typically demonstrates a spindle celled appearance but they can be epithelioid or of mixed cellular heterogenicity.

Macroscopically, these tumours are usually grey-white in appearance arising from the muscularis propria and grow either into the peritoneum (exophytically) like in this case or into the lumen of the gut (endophytically) [11]. Hirota et al. in 1998 demonstrated that GISTs arose from the interstitial cells of Cajal. These cells act as the pacemaker cells of the gut and are responsible for the peristaltic waves of the intestinal tract. Staining is also positive for CD117 (KIT oncogene and transmembrane tyrosine kinase) in over 95% of GISTs suggesting the same primitive mesenchymal cell origin thus differentiating these tumours from true leiomyomas [3, 7]. More recently, the *characteristic* expression of CD34 and CD117 (c-kit) proteins has been demonstrated [1, 7]. One paper additionally reports that the antiapoptotic factor Bcl-2 is strongly expressed in 90% of tumours with CD34 expression in as much as 78% of cases [2]. 

Unlike carcinomas, GISTs do not widely infiltrate at the microscopic level and rarely metastasize to lymph nodes or liver [1, 7]. Benign and malignant tumours differ in relation to size, presence or absence of the epithelioid component, atypia, mitotic activity, and presence or absence of tumour necrosis [2]. 

Fletcher et al. proposed a risk stratification system for GISTs which classified them according to tumour size and mitotic count which is summarised in Table 1 [10]. Eighty-five percent of tumours were larger than 6 cm metastasise compared to only 20% of those smaller than 6 cm [7, 11]. The presence of less than five mitoses per 50 high power fields (HPF) is considered low grade, whereas more than five mitoses per 50 HPF are high-grade tumours, and at much higher risk of recurrence [7, 11]. Duodenal GISTs less than 5 cm in diameter and with a mitotic index of less than 5/50 HPF carry a low risk of 8.3% for disease progression [12]. 

 Chiarugi et el. reviewed 156 duodenal GISTs in adult and paediatric patients finding that 86% of those with a tumour >5 cm with >5 mitoses per 50 HPF died of the disease, whereas no recurrence or metastases as seen in patients with tumour <2 cm with <5 mitoses per 50 HPF [5]. However, they occasionally observed the development of metastases even if the mitotic activity was <5/50 HPF and the tumour size was <5 cm [5]. Necrosis has been observed in 74.2% of malignant GISTs and was one of the features our patient did not demonstrate [2]. 

Metastases outside the abdominal cavity, such as the lung, bone, or brain, are quite rare especially as an initial presentation [7]. However, it is reported that approximately 30% of all GIST tumours will lead to local recurrence and metastases [3]. High risk malignant GISTs are more likely to lead to local recurrences, and during surgery intraperitoneal seeding and haemorrhage of the tumour can occur.

 The results of these studies would suggest that the patient in the case reported herein was at low risk for local recurrence and disease progression.

As a result of the introduction of anti-KIT tyrosine kinase inhibitors in their treatment, these tumours have become better known. Imatinib mesylate (Gleevec) is one such inhibitor that is particularly effective against the KIT protein becoming second-line treatment or neoadjuvant treatment. Up to 50% of patients with advanced disease show a response to imatinib, and for large poorly positioned tumours that are difficult to resect or unresectable, neoadjuvant treatment is recommended [8, 11]. 

There is ongoing controversy regarding optimal surgical treatment with some arguing that pancreaticoduodenectomy provides better oncological control for those located near the ampulla of Vater, and others support the selective use of a limited resection of the duodenum [3]. It is known that wide margins confer no additional benefit as there is minimal intramural spread and similarly lymphadenectomy is not routinely necessary as local lymph nodes are rarely involved. Extended resections appear to offer no additional advantage over limited wedges [7, 11]. What surgeons do agree on is that complete en bloc surgical resection is the gold standard for localized gastrointestinal tumours [8]. This offers the only curative option especially with most recurrences occurring within 2 years of identification of the original tumour [11]. To achieve this wedge, resections of the stomach or segmental resections of small intestine are usually sufficient.

For duodenal GIST, the limited wedge resection may be difficult to achieve due to proximity of adjacent structures, particularly when the tumour has extended through the serosa, and as these are uncommon locations, optimal surgical treatment is even less defined. Similar to our patient, the recent Chung et al. series support the use of wedge resections and primary closure as their most commonly performed operation [6]. 

A case series of 15 patients by Goh et al. showed comparable oncological results in patients treated with limited resection versus those treated by pancreaticoduodenectomy thus suggesting that a more extensive resection is not indicated [13]. 

Two series by Miettinen et al. reported 20% and 40%, respectively, of patients undergoing pancreatico-duodenectomy. These should be considered when localised to the second portion of the duodenum and involving complex anatomy like the ampulla [4]. If preservation of the ampulla of Vater is an issue, then segmental resection of the duodenum with duodenojejunostomy can be performed particularly for larger tumours, otherwise, limited wedge resections should be promoted particularly for those on the anterior aspect of the bowel. It is important to realise that surgery is not always curative for GISTs. Those with a primary location outside the stomach are more likely to recur, independent of size and grade [7].

As discussed, surgical resection may be achieved through several means but we suggest that limited resections should be considered even for relatively large tumours—up to 5 cm as in the present case. Segmental resection and local wedge resection were performed in the Miettinen series in 45% and 20%, respectively, knowing that only 30% of duodenal GISTs show a malignant appearance [3, 4]. 

This case highlights the successful emergency surgical management of a rare tumour with an emphasis on bowel-preservation surgery. 

## 4. Conclusion

GISTs are a rare type of gastrointestinal tumours that are most commonly located in the small bowel or stomach but can also be found in the duodenum. They tend to present with vague non-specific abdominal symptoms but occasionally will present with severe acute bleeding. We present a case of a patient with a duodenal GIST that presented with a life-threatening upper-GI bleed requiring extensive intravascular volume replacement and emergency surgery.

The most definitive treatment for GISTs is complete surgical resection which is normally achieved by segmental resection or wedge resection. Tumours of the periampullary region may require a more extensive surgical resection. The patient described herein was found to have a GIST of 5 cm in diameter on the anterior aspect of the duodenum at the junction of D3 and D4 that showed no evidence of local spread and was successfully treated with wedge resection preserving the ampulla of Vater. 

This is a preferable alternative to duodenopancreatectomy with lower morbidity and mortality but comparable oncological outcome. 

## Figures and Tables

**Table 1:** Risk of aggressive behaviour in GISTs (adapted from Fletcher et al., 2002) [10].

	Size	Mitotic count (mitoses per 50 high powered fields)
Very low risk	<2 cm	<5
Low risk	2–5 cm	<5
Intermediate risk	<5 cm	6–10
	5–10 cm	<5
High risk	>5 cm	>5
	>10 cm	Any mitotic rate
	Any size	>10

## References

[1] Liyanage CAH, Abeygunawardhana S, Kumarage S, Deen KI (2008). Duodenum-preserving local excision of a gastrointestinal stromal tumor. *Hepatobiliary and Pancreatic Diseases International*.

[2] Rabin I, Chikman B, Lavy R (2009). Gastrointestinal stromal tumors: a 19 year experience. *The Israel Medical Association Journal*.

[3] Mennigen R, Wolters HH, Schulte B, Pelster FW (2008). Segmental resection of the duodenum for gastrointestinal stromal tumor (GIST). *World Journal of Surgical Oncology*.

[4] Miettinen M, Kopczynski J, Makhlouf HR (2003). Gastrointestinal stromal tumors, intramural leiomyomas, and leiomyosarcomas in the duodenum: a clinicopathologic, immunohistochemical, and molecular genetic study of 167 cases. *American Journal of Surgical Pathology*.

[5] Chiarugi M, Galatioto C, Lippolis P, Zocco G, Seccia M (2007). Gastrointestinal stromal tumour of the duodenum in childhood: a rare case report. *BMC Cancer*.

[6] Chung JC, Chu CW, Cho GS (2010). Management and outcome of gastrointestinal stromal tumours of the duodenum. *Journal of Gastrointestinal Surgery*.

[7] Chaudhry UI, DeMatteo RP (2011). Advances in the surgical management of gastrointestinal stromal tumor. *Advances in Surgery*.

[8] Hassani K, Zahid FZ, Ousadden A, Mazaz K, Ait Taleb K (2009). Gastrointestinal stromal tumours and shock. *Journal of Emergencies, Trauma and Shock*.

[9] Aboutaleb E, Kothari M, Damrah O, Canelo R (2009). C-KIT positive gastrointestinal stromal tumor presenting with acute bleeding in a patient with neurofibromatosis type 1: a case report. *International Seminars in Surgical Oncology*.

[10] Fletcher CD, Berman JJ, Corless C (2002). Diagnosis of gastrointestinal stromal tumors: a consensus approach. *Human Pathology*.

[11] Towu E, Stanton M (2006). Gastrointestinal stromal tumour presenting with severe bleeding: a review of the molecular biology. *Pediatric Surgery International*.

[12] Miettinen M, Lasota J (2006). Gastrointestinal stromal tumors: review on morphology, molecular pathology, prognosis, and differential diagnosis. *Archives of Pathology and Laboratory Medicine*.

[13] Goh BKP, Chow PKH, Kesavan S, Yap WM, Wong WK (2008). Outcome after surgical treatment of suspected gastrointestinal stromal tumors involving the duodenum: is limited resection appropriate?. *Journal of Surgical Oncology*.

